# Prognostic implications of microRNA-107 in esophageal cancer: a retrospective cohort study

**DOI:** 10.7717/peerj.20327

**Published:** 2025-11-06

**Authors:** Jie Sun, Jin Zhang, Jingwen Ye, Yuqi Chen, Caifeng Jiang

**Affiliations:** 1 Department of Gastroenterology, The Fourth Affiliated Hospital of Soochow University, Suzhou, Jiangsu, China; 2 Department of Pathology, Suzhou Hospital, Affiliated Hospital of Medical School, Nanjing University, Suzhou, Jiangsu, China; 3 Department of Pathology, Nanjing General Hospital of Nanjing Military Command, Nanjing, Jiangsu, China

**Keywords:** Esophageal squamous cell carcinoma, MiR-107, Prognostic biomarker, Survival analysis, Molecular staging

## Abstract

**Background:**

Esophageal squamous cell carcinoma (ESCC) imposes a heavy disease burden in China, accounting for over 50% of global cases and approximately 301,000 annual deaths. Current prognostic markers inadequately predict recurrence in early-stage patients. This study investigates microRNA-107 (miR-107) as a novel prognostic biomarker for ESCC.

**Methods:**

Tumor tissues (*n* = 66) and adjacent normal tissues (*n* = 28) were collected from ESCC patients undergoing radical surgery (2010–2012). miR-107 expression was quantified *via* reverse transcription quantitative polymerase chain reaction (RT-qPCR) (normalized to U6 snRNA). Clinicopathological correlations and survival outcomes were analyzed using *χ*^2^ tests, Kaplan–Meier/log-rank tests, and Cox regression. Comparative analysis of miR-107 levels was performed in human esophageal squamous cell carcinoma line 109 (EC109) cancer cells versus human esophageal epithelial cell (HEEC) normal epithelial cells.

**Results:**

miR-107 expression was significantly lower in esophageal cancer tissues (0.801 ± 0.737) compared to adjacent non-cancerous tissues (1.390 ± 1.346), *p* = 0.006. Low miR-107 expression (cutoff = median) correlated with advanced tumor, node, metastasis stage (TNM stage) (I *vs.* V: 100% *vs.* 21.4%, *P* < 0.001), lymph node metastasis (73.1% *vs.* 35%, *P* < 0.001), and larger tumor size (70% *vs.* 33.3%, *P* < 0.001). Patients with low miR-107 had shorter median overall survival (10 *vs.* 59 months; Hazard Ratio (HR) = 0.475, 95% Confidence Interval (CI) [0.247–0.915]; *P* < 0.001). Multivariate Cox analysis confirmed miR-107 as an independent prognostic factor alongside TNM stage (HR = 3.586, 95% CI [2.253–5.708]; *P* < 0.001). Consistently, EC109 cells exhibited 59% lower miR-107 levels than HEEC (*P* = 0.029).

**Conclusions:**

miR-107 downregulation is a robust predictor of aggressive ESCC phenotypes and poor survival. It holds promise as a clinical biomarker for risk stratification and personalized therapy. Future studies should validate these findings in multicenter cohorts and elucidate miR-107’s functional mechanisms.

## Introduction

Esophageal cancer (EC) ranks as the seventh most prevalent malignancy globally, with an estimated 604,000 new cases and 544,000 deaths annually according to Global Cancer Observatory 2022 (GCO 2022) (Bray et al., 2024). China bears a disproportionate burden, accounting for over 50% of global EC incidence (322,000 cases/year) and mortality (301,000 deaths/year), making it the fourth leading cause of cancer-related deaths nationwide (Han et al., 2024). Notably, esophageal squamous cell carcinoma (ESCC) constitutes 90% of Chinese EC cases, with endemic hotspots in Henan, Hebei, and Shanxi provinces—regions where age-standardized incidence rates exceed 100/100,000, linked to chronic exposure to dietary carcinogens (nitrosamine-rich preserved foods, high-temperature beverages), tobacco use, and alcohol consumption (Wei et al., 2020; Abnet, Arnold & Wei, 2018).

Despite multimodal therapies (surgery, chemoradiotherapy), 5-year survival rates remain dismal (15–25%), largely due to late-stage diagnosis and inadequate prognostic stratification (Turco, Donzelli & Fontemaggi, 2020). Current staging systems (American Joint Committee on Cancer (AJCC) TNM 8th edition) rely on clinicopathological features (tumor size, lymph node status), yet fail to predict recurrence in 30–40% of early-stage (I–II) patients post-resection (Kröll et al., 2018). This underscores the urgent need for molecular biomarkers to refine risk stratification and guide adjuvant therapy.

While conventional ESCC biomarkers (*e.g.*, squamous cell carcinoma antigen (SCC-Ag), cytokeratin 19 fragment (CYFRA 21-1)) exhibit limited early-stage sensitivity (sensitivity < 60% for Stage I/II tumors) (Nakamura et al., 2017; Quillien et al., 1998), novel miRNA-based markers have emerged as promising alternatives. Among these, miR-107—a conserved miRNA located on chromosome 10q23.31—exhibits context-dependent roles across cancers. In ESCC, preclinical studies report tumor-suppressive functions *via* targeting oncogenic pathways (*e.g.*, cell division cycle 42 (Cdc42)-mediated invasion (Sharma, Saini & Sharma, 2017), epidermal growth factor receptor/phosphatidylinositol 3-kinase/ak strain transforming (EGFR/PI3K-AKT) signaling (Wei et al., 2024)). Despite compelling preclinical evidence, clinical validation of miR-107 in ESCC remains critically sparse. To date, fewer than five studies have correlated its expression with survival outcomes (Sharma, Saraya & Sharma, 2018; Zheng et al., 2017), and none have integrated long-term follow-up with cell-based mechanistic validation. This study bridges this gap by investigating miR-107’s prognostic utility in a well-characterized ESCC cohort (*n* = 66 with 8-year follow-up), combining clinicopathological analysis, survival modeling, and *in vitro* functional correlation to establish its clinical relevance.

## Materials & Methods

### Clinical information and sample collection

#### Study design and ethical statement

This retrospective cohort study was approved by the Ethics Committee of The Fourth Affiliated Hospital of Soochow University (No. 251178), and verbal informed consent was obtained from all participants prior to their inclusion in this study.

### Patient enrollment and sample collection

Tumor tissues (*n* = 66) and paired adjacent non-cancerous tissues (*n* = 28) were collected from ESCC patients who underwent radical esophagectomy at Nanjing General Hospital of Nanjing Military Command between January 2010 and December 2012. Inclusion criteria: (1) Pathologically confirmed ESCC; (2) no preoperative radiotherapy/chemotherapy; (3) complete resection (R0) with no distant metastasis; (4) availability of complete clinicopathological data (gender, age, tumor size, tumor, node, metastasis (TNM) stage (AJCC 8th edition), lymph node status, *etc.*). Exclusion criteria: (1) Non-squamous histology (*e.g.*, adenocarcinoma); (2) incomplete clinical records; (3) perioperative mortality (death within 30 days post-surgery).

### Follow-up strategy

Patients were followed every 3 months for the first 2 years and every 6 months thereafter until December 2020. Overall survival (OS) was defined as the interval from surgery to death or last follow-up. Of 66 patients, 50 died during follow-up.

### Rationale for grouping thresholds

**Tumor size cutoff (three cm)**: The tumor size cutoff of three cm was selected based on the TNM staging criteria for esophageal cancer (AJCC 8th edition), where tumors >three cm are associated with advanced T2/T3 stages and poorer prognosis. This threshold has been widely adopted in previous studies investigating tumor size as a prognostic factor (Amin et al., 2017).

**Age cutoff (60 years)**: The age cutoff of 60 years was chosen based on two considerations: (1) It aligns with the median age of the cohort (median = 59 years), allowing balanced subgroup comparisons; (2) Previous epidemiological studies have identified 60 years as a critical threshold for increased treatment resistance and mortality in esophageal cancer patients (Xia et al., 2022).

### Experimental materials and methods

#### Reagents and instruments

RNA extraction from tissues and cell lines (human esophageal squamous cell carcinoma Line 109 (EC109), human esophageal epithelial cell (HEEC)) was performed using a column-based miRNA isolation kit (B518811; Sango Biotech, Shanghai, China). First-strand cDNA synthesis was carried out with a stem-loop RT primer-based miRNA cDNA synthesis kit (B532453; Sango Biotech), using one µg total RNA input as quantified by NanoDrop 2000 spectrophotometer (Thermo Fisher Scientific; A260/A280 ratio: 1.8–2.1, A260/A230 ratio: ≥1.5). Quantitative polymerase chain reaction (qPCR) amplification utilized SYBR Green Pro Taq HS premix (AG11701; Accurate Biology, Hunan, China) containing ROX passive reference dye, with reactions sealed by Axygen PlateMax Ultraclear film (UC-500; Corning, NY, USA). Thermal cycling was performed on a QuantStudio™ 3 Real-Time PCR System (Thermo Fisher Scientific) with a 96-well block, and plates were centrifuged using a XiangYi L-530 microplate centrifuge (Hunan XiangYi Laboratory Instrument Co.). Primer sequences (synthesized by Sango Biotech, Shanghai, China) were designed using miRBase (Release 22) for miR-107 and RNU6-1 (U6) snRNA, with stem-loop RT primer specificity validated by BLAST against the human genome (NCBI). Primer details:

miR-107:

stem-loop primer: 5′- GTCGTATCCAGTGCAGGGTCCGAGGTATTCGCACTGGAT ACGACTGATAG-3′ (10 µM stock)

Forward: 5′- CAAGCCGAGCAGCATTGTACAG-3′ (10 µM, Tm = 58 °C)

Reverse: 5′- ATCCAGTGCAGGGTCCGAGG-3′ (10 µM)

U6 snRNA

Forward: 5′-CTCGCTTCGGCAGCACA-3′ (10 µM, Tm = 60 °C)

Reverse: 5′-AACGCTTCACGAATTTGCGT-3′ (10 µM).

#### miRNA extraction and qPCR

Total RNA integrity was confirmed by 1% agarose gel electrophoresis (28S/18S rRNA ratio > 1.5) , with concentration and purity measured *via* NanoDrop (minimum 100 ng/µL; A260/A280 ratio 1.8–2.1). Absence of genomic DNA contamination was verified by no-template controls. cDNA synthesis reactions (20 µL) contained one µg RNA, 50 U Maxima H Minus Reverse Transcriptase, and one µM stem-loop RT primer, incubated at 16 °C (30 min), 37 °C (30 min), 85 °C (5 min; inactivation). qPCR amplification (20 µL) consisted of 10 µL SYBR Green mix (2× concentration), one µL cDNA template (diluted 1:5), and 0.5 µM each forward/reverse primer. Cycling conditions: 95 °C for 30 s (initial denaturation), followed by 40 cycles of 95 °C for 5 s and 60 °C for 30 s (annealing/extension). Melting curve analysis (60–95 °C, 0.3 °C/s increment) confirmed single amplicon specificity. Three biological replicates (independent RNA extracts) with triplicate technical repeats were analyzed. Samples with Ct values >35 cycles for either miR-107 or U6 were excluded. Amplification efficiencies (E = 98–105%) were determined *via* standard curve (10-fold serial dilutions; *R*^2^ > 0.99), U6 snRNA expression stability was validated by geNorm (M-value < 0.5 across tissue and cell samples), and relative miR-107 expression was calculated using the 2^−ΔΔCt^ method (Livak & Schmittgen, 2001).

#### Cellular validation of miR-107 expression

The human esophageal epithelial cell line HEEC (BNCC340554) and esophageal squamous cell carcinoma line EC109 (BNCC337680) were obtained from Beina Chuanglian Biotechnology Co., Ltd. (Beijing, China). Cells were cultured in RPMI-1640 medium (Gibco) supplemented with 10% fetal bovine serum (FBS; Biological Industries) and 1% penicillin/streptomycin (Beyotime) at 37 °C in a 5% CO_2_ humidified incubator. Upon reaching 80–90% confluency, cells were harvested using 0.25% trypsin-EDTA. (Beyotime) RNA extraction, quality control, and qPCR protocols strictly followed ‘miRNA Extraction and qPCR’, including equivalent RNA input (one µg) and matching primer concentrations. cDNA synthesis and reverse transcription quantitative polymerase chain reaction (RT-qPCR) amplification were performed with U6 snRNA as the endogenous control. All experiments included three biological replicates, each run in technical triplicate. The comparative 2^−ΔΔCt^ method was used to quantify miR-107 expression in EC109 cells relative to HEEC controls.

### Statistical analysis

All statistical analyses were conducted using SPSS 29.0 (IBM Corp., Armonk, NY, USA) and R 4.3.0 (R Core Team, 2023; survminer package for maximally selected log-rank statistics). Normality of continuous variables was assessed using the Shapiro–Wilk test (for samples < 50) or Kolmogorov–Smirnov test (for samples ≥ 50). Variables following a normal distribution were compared using independent samples *t*-tests, while non-normally distributed variables were analyzed with the Mann–Whitney U test. Categorical variables were compared *via* Pearson’s chi-square test or Fisher’s exact test, as appropriate.

For survival analysis, the optimal cutoff value for miR-107 expression (continuous variable) was determined using maximally selected log-rank statistics, which identifies the threshold that maximizes the survival difference between subgroups. Survival curves were generated using the Kaplan–Meier method, and differences were assessed with the log-rank test. Variables showing a trend toward significance in univariate analysis (*P* < 0.1) were included in a forward stepwise Cox proportional hazards regression model (entry: α = 0.05; removal: α = 0.1). The proportional hazards assumption was confirmed using Schoenfeld residuals.

Data are presented as mean ± standard deviation (normally distributed) or median (interquartile range) (non-normal). Hazard ratios (HRs) are reported with 95% confidence intervals (CIs). A two-tailed *P* < 0.05 was considered statistically significant.

## Results

### miR-107 expression in tumor *vs.* adjacent non-cancerous tissues

RT-qPCR analysis revealed significantly lower miR-107 expression in esophageal cancer tissues (*n* = 66, mean ± SD: 0.801 ± 0.737, 2^−ΔΔCt^) compared to adjacent non-cancerous tissues (*n* = 28, mean ± SD: 1.390 ± 1.346; *P* = 0.006, Mann–Whitney *U* test) (Table 1). The non-normal distribution of miR-107 expression in tumor tissues (Kolmogorov–Smirnov test: *Z* = 0.241, *P* < 0.001) justified the use of the median value (0.652) as the cutoff for subsequent survival analyses.

**Table 1:** miR-107 expression levels in esophageal cancer tissues and adjacent non-cancerous tissues.

Tissue type	*N*	Mean expression (2^−ΔΔCT^)	*Z*-Value	*p*-Value	Exact *p*-value
Esophageal cancer	66	0.801 ± 0.737			
Adjacent non-cancerous	28	1.390 ± 1.346	−2.724	0.006	0.006

### Survival analysis based on miR-107 expression

**Figure 1:**
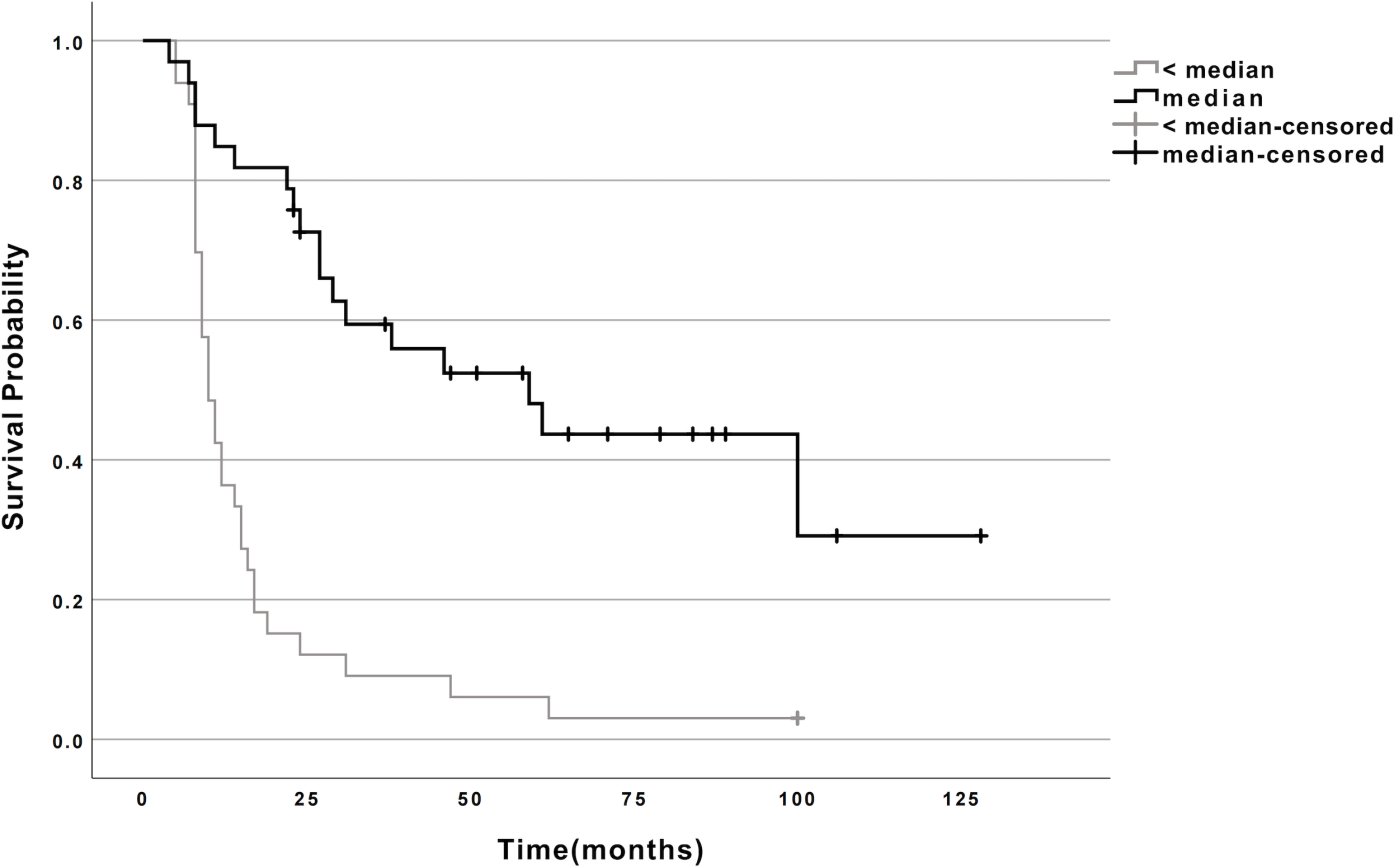
Survival curves for esophageal cancer patients with high and low miR-107 expression. Survival curves for esophageal cancer patients categorized by miR-107 expression levels. The curves illustrate the survival probability over time for patients with high miR-107 expression compared to those with low miR-107 expression. Note: ≥ median: Patients with miR-107 expression at or above the median; < median: Patients with miR-107 expression below the median.

Using maximally selected log-rank statistics, the median miR-107 expression (0.652) optimally stratified patients into high- (*n* = 33) and low-expression (*n* = 33) groups. Kaplan–Meier analysis demonstrated a striking survival advantage for the high-expression group: (1) Median survival: 59 months (95% CI [24.9–93.1]) *vs.* 10 months (95% CI [7.7–12.3]) in the low-expression group (log-rank *P* < 0.001; Fig. 1, Table 2). (2) Mortality rates: 54.5% (18/33) *vs.* 97.0% (32/33), respectively.

**Table 2:** Log-rank univariate analysis of survival time and miR-107 expression levels in esophageal cancer patients. Statistical significance was assessed using the log-rank test, with a *p*-value < 0.05, indicating a significant difference in survival times based on miR-107 expression levels.

miR-107	*N*	Deaths	CENSORED		Median Survival Time (Months)		95% CI	*χ*2	*P*
			*N*	Survival rate (%)				
<median	33	32	1	3	10	7.749∼12.251			
≥median	33	18	15	45.5	59	24.921∼93.079		25.598	<0.001

### Association between miR-107 expression and clinicopathological features

miR-107 expression showed no significant correlation with age, gender, tumor location, histological type, p53 mutations, vascular invasion, or neural invasion (*P* > 0.05 for all). However, it was strongly associated with: (1) TNM stage: Expression rates decreased progressively from Stage I (100%) to Stage IV (21.4%) (*P* < 0.001, *χ*^2^ test for trend). (2) Lymph node metastasis: 73.1% *vs.* 35.0% in N0 *vs.* N+ cases (*P* < 0.001. (3) Tumor size: ≤3 cm tumors exhibited higher expression (70.0%) compared to >3 cm tumors (33.3%; *P* < 0.001) (Table 3).

**Table 3:** Association between miR-107 expression and clinical pathological characteristics in esophageal cancer tissues. *P* values calculated by *χ*^2^ test or Fisher’s exact test. Tumor size cutoff: 3 cm (AJCC TNM staging criteria). The age threshold of 60 years was selected based on the cohort’s median age and epidemiological data.

Clinical data	miR-107			
(N)	<median (N,%)	≥median (N,%)	*χ*2	*p*
**Age**				
<60 (*n* = 40)	18 (45)	22 (55)		
≥60 (*n* = 26)	15 (57.7)	11 (42.3)	1.100	0.294
**Gender**				
Male (*n* = 44)	22 (50)	22 (50)		
Female (*n* = 22)	11 (50)	11 (50)	0.003	0.958
**Tumor location**				
Thoracic esophagus (*n* = 57)	29 (50.9)	28 (49.1)		
Abdominal esophagus (*n* = 9)	4 (44.4)	5 (55.6)	0.075	0.784
**Gross classification**				
Ulcerative (*n* = 43)	23 (53.5)	20 (46.5)		
Medullary (*n* = 14)	5 (35.7)	9 (64.3)		
Constrictive (*n* = 7)	3 (42.9)	4 (57.1)		
Fungous (*n* = 2)	2 (100)	0 (0)	1.499	0.683
**p53 mutation**				
Mutation (*n* = 23)	14 (60.9)	9 (39.1)		
Wild-type (*n* = 43)	19 (44.2)	24 (55.8)	2.399	0.121
**TNM stage**				
I (*n* = 4)	0 (0)	4 (100)		
II (*n* = 22)	3 (13.6)	19 (86.4)		
III (*n* = 26)	19 (73.1)	7 (26.9)		
IV (*n* = 14)	11 (78.6)	3 (21.4)	45.557	<0.001
**Vascular invasion**				
Present (*n* = 7)	3 (42.9)	4 (57.1)		
Absent (*n* = 59)	30 (50.8)	29 (49.2)	0.263	0.608
**Nerve invasion**				
Present (*n* = 8)	4 (50)	4 (50)		
Absent (*n* = 58)	29 (50)	29 (50)	0.389	0.533
**Lymph node metastasis**				
Present (*n* = 40)	26 (65)	14 (35)		
Absent (*n* = 26)	7 (26.9)	19 (73.1)	11.541	<0.001
**Tumor size**				
<3 cm (*n* = 30)	9 (30)	21 (70)		
≥3 cm (*n* = 36)	24 (66.7)	12 (33.3)	14.145	<0.001

### Multivariate cox proportional hazards analysis

Forward stepwise Cox regression (adjusting for variables with *P* < 0.1 in univariate analysis) identified two independent prognostic factors: (1) miR-107 high expression: Protective effect (HR = 0.475, 95% CI [0.247–0.915]; P = 0.026). (2) Advanced TNM stage: Increased mortality risk (HR = 3.586, 95% CI [2.253–5.708]; *P* < 0.001) (Table 4).

**Table 4:** Cox regression analysis of prognostic factors in esophageal cancer patients.

Prognostic factor	*β*	SE	Wald	*P*	HR	95% CI
TNM stage	1.277	0.237	29.010	<0.001	3.586	2.253∼5.708
miR-107	−0.744	0.334	4.953	0.026	0.475	0.247∼0.915

### miR-107 expression in EC109 *vs.* HEEC cells

**Figure 2:**
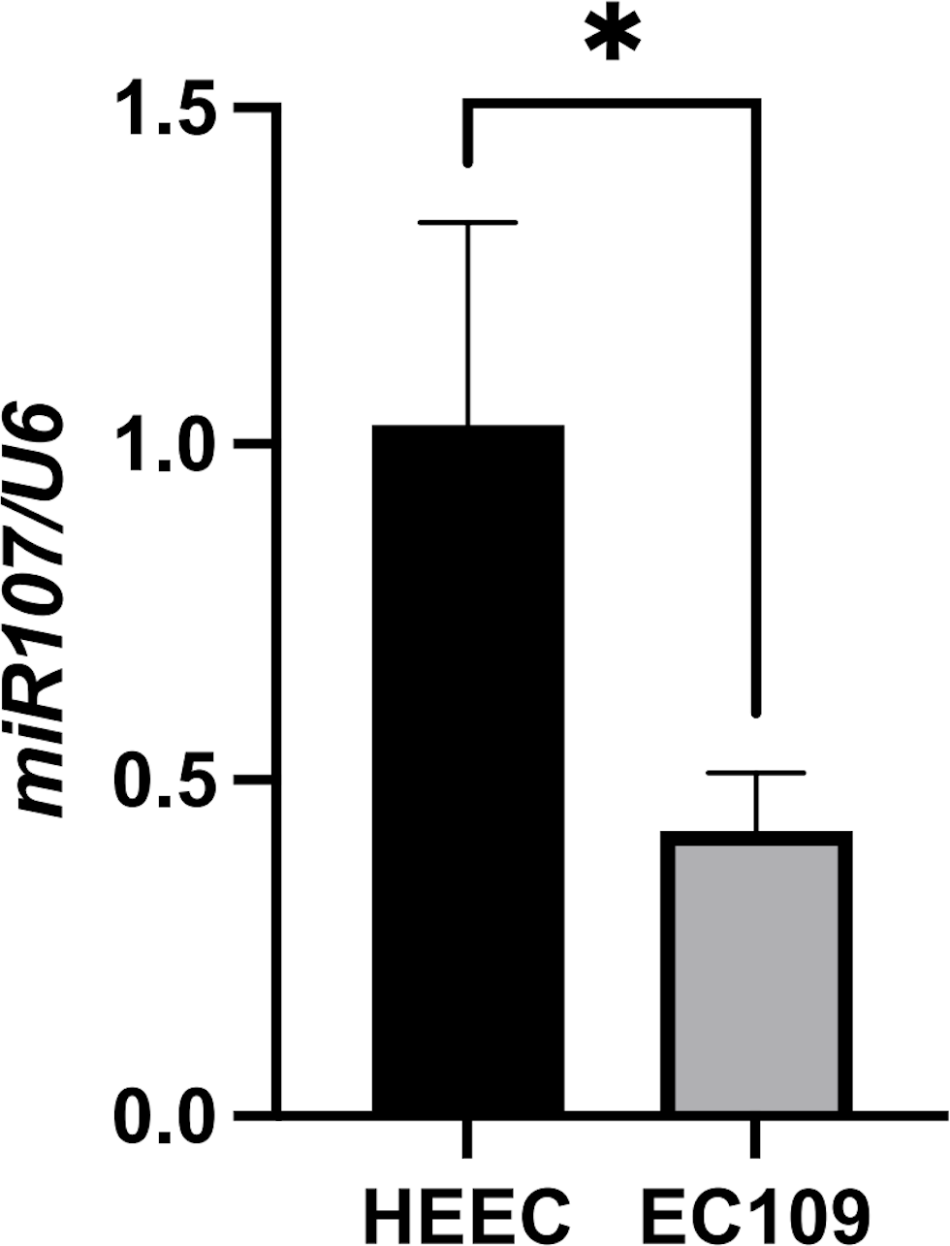
Comparison of miR-107 expression levels between EC109 esophageal cancer cells and normal HEEC cells. Differential expression of miR-107 in EC109 esophageal cancer cells compared to normal HEEC esophageal epithelial cells (EC109 *vs.* HEEC cells, *p* = 0.029).

Consistent with clinical findings, miR-107 expression was significantly reduced in EC109 esophageal cancer cells (mean ± SD: 0.425 ± 0.086, 2^−ΔΔCt^) compared to normal HEEC epithelial cells (mean ± SD: 1.028 ± 0.300, 2^−ΔΔCt^) (fold change = 0.41, *P* = 0.029, unpaired *t*-test) (Fig. 2), miR-107 downregulation in EC109 cells reinforces its tumor-suppressive role in ESCC pathogenesis.

## Discussion

Our study establishes miR-107 as a key regulator in esophageal cancer progression and prognosis. The significant downregulation of miR-107 in tumor tissues and EC109 cells (*vs.* HEEC, Fig. 2), coupled with its strong association with survival (HR = 0.475, Table 4), underscores its tumor-suppressive role. Mechanistically, prior studies in ESCC suggest that miR-107 targets oncogenic pathways including Cdc42-mediated cytoskeletal dynamics (Sharma, Saini & Sharma, 2017) and EGFR/PI3K/AKT signaling (Wei et al., 2024), which aligns with our observation of reduced miR-107 in advanced TNM stages and metastatic cases (Table 3).

The dual role of miR-107 in other cancers—suppressing metastasis in breast cancer *via* neural precursor cell expressed, developmentally down-regulated 9 (NEDD9) (Zhou et al., 2022) yet promoting growth in non-small cell lung cancer (NSCLC) *via* large tumor suppressor kinase 2/yes-associated protein (LATS2/YAP) (Jin et al., 2020)—highlights the necessity of context-specific validation. In ESCC, our findings and existing evidence (Sharma, Saini & Sharma, 2017; Chang et al., 2021; Zhang et al., 2022) consistently demonstrate miR-107’s tumor-suppressive function. Mechanistically, miR-107 directly binds the 3′-UTR of Tropomyosin 3 (TPM3), suppressing this oncogenic driver and subsequently inhibiting matrix metalloproteinase 2/matrix metalloproteinase 9 (MMP2/MMP9)-mediated epithelial-mesenchymal transition (EMT), thereby curbing proliferation, invasion, and metastasis. Clinically, miR-107 is significantly downregulated in ESCC tissues (particularly advanced stages), correlating with poor differentiation, lymph node metastasis, and adverse prognosis—a pattern reinforced by rescue experiments showing TPM3 overexpression reverses miR-107–mediated suppression. Epigenetic silencing *via* promoter hypermethylation further consolidates its context-dependent tumor-suppressive activity in ESCC pathogenesis.

Clinically, miR-107 may serve as a complementary prognostic biomarker to conventional ESCC staging (*e.g.*, TNM and nodal status), particularly in Stage II-III tumors where its low expression correlates with reduced metastasis-free survival. This potential contrasts with its oncogenic role in gastric cancer *via* targeting Dicer 1, Ribonuclease III (DICER1) and Phosphatase and TENsin homolog (PTEN) (Ren et al., 2019; Wang et al., 2019) highlighting tissue-specific functionality. Standardized validation (*e.g.*, RT-qPCR with miR-16 normalization) remains essential before clinical translation, given the limited sensitivity of current serum markers like the carcinoembryonic antigen (CEA) (45–60%) for tissue miRNA assessment.

To ensure robust survival analysis, we utilized samples collected during 2010–2012 with at least complete 8-year follow-up (until 2020). This design is critical for ESCC prognosis studies, where >50% of deaths occur beyond 3 years post-diagnosis (Yang et al., 2021). The extended observation period enabled accurate assessment of miR-107’s prognostic value, while uniform sample processing minimized technical variability—a key confounder in miRNA biomarker research (Mitchell & Searles, 2020).

Despite these insights, our study has limitations. To address generalizability concerns, we propose validating miR-107 in external cohorts such as The Cancer Genome Atlas (TCGA) ESCC dataset (*n* = 95) or prospective registries (*e.g.*, oesophageal cancer clinical and molecular stratification (OCCAMS) consortium). The retrospective design and modest sample size (*n* = 66) limit statistical power for rare subgroups (*e.g.*, p53 mutant cases). Future multicenter cohorts should validate miR-107’s prognostic utility, incorporating standardized pre-analytical protocols for miRNA quantification. Critically, the absence of functional data represents a key gap. We propose a systematic approach to delineate miR-107s mechanisms: (1) *in vitro* modulation (mimics/antagomirs) to assess proliferation, invasion, and chemoresistance; (2) RNA-seq and CLIP-seq to identify direct targets; (3) *in vivo* validation using orthotopic ESCC models.

## Conclusions

In conclusion, miR-107 represents a promising prognostic biomarker candidate in ESCC. Future validation in multi-center cohorts (*e.g.*, integrating TCGA-ESCC data) is essential before clinical translation. Mechanistic studies should clarify its tissue-specific roles, potentially informing targeted therapies for high-risk patients.

## Supplemental Information

10.7717/peerj.20327/supp-1 Supplemental Information 1 ESCC miR107 Clinical Data Statistical Results

10.7717/peerj.20327/supp-2 Supplemental Information 2 MIQE Checklist

## Additional Information and Declarations

### Data Availability

The following information was supplied regarding data availability: The raw measurements are available in the Supplemental Files. The data is available at Zenodo: sun, jie. (2025). Clinical and qPCR raw data: miR-107 as a prognostic biomarker in esophageal squamous cell carcinoma (ESCC) [Data set]. Zenodo. https://doi.org/10.5281/zenodo.16197896
